# Heterogeneity of Regional Brain Atrophy Patterns Associated with Distinct Progression Rates in Alzheimer’s Disease

**DOI:** 10.1371/journal.pone.0142756

**Published:** 2015-11-30

**Authors:** Min Soo Byun, Song E. Kim, Jinsick Park, Dahyun Yi, Young Min Choe, Bo Kyung Sohn, Hyo Jung Choi, Hyewon Baek, Ji Young Han, Jong Inn Woo, Dong Young Lee, Alzheimer’s Disease Neuroimaging Initiative

**Affiliations:** 1 Department of Neuropsychiatry, Seoul National University Hospital, Seoul, Republic of Korea; 2 Department of Biomedical Engineering, Hanyang University, Seoul, Republic of Korea; 3 Department of Neuropsychiatry, Ulsan University Hospital, Ulsan, Republic of Korea; 4 Department of Neuropsychiatry, Seoul Metropolitan Government-Seoul National University Boramae Medical Center, Seoul, Republic of Korea; 5 Neuroscience Research Institute, Medical Research Center Seoul National University, Seoul, Republic of Korea; Sungkyunkwan University, REPUBLIC OF KOREA

## Abstract

We aimed to identify and characterize subtypes of Alzheimer’s disease (AD) exhibiting different patterns of regional brain atrophy on MRI using age- and gender-specific norms of regional brain volumes. AD subjects included in the Alzheimer's Disease Neuroimaging Initiative study were classified into subtypes based on standardized values (Z-scores) of hippocampal and regional cortical volumes on MRI with reference to age- and gender-specific norms obtained from 222 cognitively normal (CN) subjects. Baseline and longitudinal changes of clinical characteristics over 2 years were compared across subtypes. Whole-brain-level gray matter (GM) atrophy pattern using voxel-based morphometry (VBM) and cerebrospinal fluid (CSF) biomarkers of the subtypes were also investigated. Of 163 AD subjects, 58.9% were classified as the “both impaired” subtype with the typical hippocampal and cortical atrophy pattern, whereas 41.1% were classified as the subtypes with atypical atrophy patterns: “hippocampal atrophy only” (19.0%), “cortical atrophy only” (11.7%), and “both spared” (10.4%). Voxel-based morphometric analysis demonstrated whole-brain-level differences in overall GM atrophy across the subtypes. These subtypes showed different progression rates over 2 years; and all subtypes had significantly lower CSF amyloid-β_1–42_ levels compared to CN. In conclusion, we identified four AD subtypes exhibiting heterogeneous atrophy patterns on MRI with different progression rates after controlling the effects of aging and gender on atrophy with normative information. CSF biomarker analysis suggests the presence of Aβ neuropathology irrespective of subtypes. Such heterogeneity of MRI-based neuronal injury biomarker and related heterogeneous progression patterns should be considered in clinical trials and practice with AD patients.

## Introduction

Clinical diagnosis of Alzheimer’s disease (AD) has generally relied on symptom-oriented criteria [1], which are both variable and insufficiently accurate [2, 3]. To enhance diagnostic accuracy, a number of studies have attempted to develop various biomarkers of AD to complement the diagnostic criteria. Recent advances in the use of biomarkers have led to new diagnostic criteria for AD, including the National Institute on Aging–Alzheimer’s Association (NIA-AA) diagnostic guidelines for AD [4, 5]. AD biomarkers fall into two major categories: biomarkers of brain β-amyloid (Aβ) protein deposition and biomarkers of neuronal injury or degeneration. Among neuronal injury biomarkers, atrophy of the hippocampus and association cortices revealed by magnetic resonance imaging (MRI) has been consistently reported, and is regarded as the typical regional pattern of brain atrophy in AD [6–9]. Such a specific pattern of atrophy aligns well with the topographic distribution of AD pathology such as neurofibrillary tangles (NFT) [10, 11] and correlates strongly with the severity of cognitive decline [6, 9, 12–15]. As reported previously [8, 16], MRI-based neuronal injury biomarkers are becoming more widely used in clinical practice as probability of having AD in subjects who meet clinical diagnostic criteria of AD [1] is estimated to increase if MRI-based neuronal injury biomarker is positive or decrease if negative. In addition, interests on using MRI-based neuronal injury biomarker to reduce sample sizes required for a new drug trial or track disease progression as an outcome measure are also increasing currently [7, 17, 18].

However, several MRI studies suggest that not all AD patients demonstrate this typical pattern of brain atrophy. For instance, 10–30% of AD patients did not exhibit typical hippocampal atrophy [7, 8, 19]. Recent neuropathological studies also found that about 25–30% of AD cases did not show the typical distribution of NFT in the hippocampus or association cortices and were classified as atypical subtypes including “hippocampal-sparing” and “limbic-predominant” AD [20, 21]. Moreover, these pathological subtypes exhibited differences in clinical characteristics including the rate of disease progression [20]. Therefore, when using MRI-based neuronal injury biomarkers to enhance diagnostic accuracy in clinical practice or to select candidates in drug trials, such heterogeneity issues should be seriously considered.

One of the challenges to use MRI-based neuronal injury biomarkers such as regional brain atrophy in research or clinical practice is a lack of normative values of MRI measurements [22]. Since regional brain atrophy can be significantly affected by normal aging, gender differences as well as by pathological processes such as AD [23, 24], any attempt to accurately identify AD subtypes using specific patterns of regional brain atrophy should employ age- and gender-specific normative values of regional brain volumes obtained from a large cognitively normal (CN) population. Although some previous studies have reported variability of regional brain atrophy patterns in AD patients using MRI measurements [25–27], there have been no studies that used norm-based approach to control the effect of normal aging and gender difference on atrophy.

We first aimed to identify subtypes of AD exhibiting heterogeneous atrophy patterns in the hippocampus and association cortices. These regions were selected based on the results of previous neuropathological studies that classified AD subtypes using the patterns of NFT distribution in the hippocampus and association cortices [20, 21]. To accurately identify such subtypes while minimizing potentially confounding effects of aging and gender difference on atrophy, we used the standardized values (Z-scores) of regional volumes based on age- and gender-specific norms derived from a large MRI dataset of a CN group in the Alzheimer’s Disease Neuroimaging Initiative (ADNI) study rather than simply adopting raw volume values. We then compared clinical characteristics across the subtypes, including longitudinal progression rates and cross-sectional features. Finally, we investigated the levels of cerebrospinal fluid (CSF) biomarkers including CSF amyloid-β 1–42 peptide (Aβ_1–42_), total tau (t-tau), and phosphorylated tau (p-tau), to describe the pathological substrates of the various subtypes.

## Materials and Methods

### Ethics Statement

Data used in this article were obtained from the ADNI database (http://adni.loni.usc.edu/), which over 50 institutions participated in. The full list of ADNI’s participating institutions can be found at http://adni.loni.usc.edu/about/centers-cores/study-sites/. Written informed consent was obtained from all participants and the study was conducted after prior Institutional Review Board approval was obtained at each participating institution. For more information, see http://www.adni-info.org/Scientists/doc/ADNI_Protocol_Extension_A2_091908.pdf.

### Participants

The data reported in this article were obtained from the ADNI database (adni.loni.usc.edu). Initially aiming to recruit 800 subjects, the ADNI-1 was subsequently followed by ADNI-GO and ADNI-2. To date these three protocols have recruited a total of over 1,500 adults, aged 55–90 years, including CN older individuals, subjects with MCI, and subjects meeting the criteria for probable AD set out by the National Institute of Neurological and Communicative Disorders and Stroke and the Alzheimer’s Disease and Related Disorders Association (NINCDS-ADRDA). Details of inclusion/exclusion criteria and protocols have been previously described [28]. For up-to-date information, see www.adni-info.org.

Data from the ADNI-1 study were downloaded in April 2013. Baseline data including demographic, clinical, neuropsychological variables, and 1.5 Tesla brain MRI scans from 398 subjects including 227 CN and 171 AD cases were downloaded. Among them, 112 CN and 87 AD subjects had baseline CSF biomarker data. In addition, 1-year and 2-year follow-up data of the neuropsychological measures were available for a subset of subjects. Number of available data for longitudinal analysis of neuropsychological measures is described in S3 Table.

### Demographic, clinical, and neuropsychological variables

Baseline demographic and clinical variables including global Clinical Dementia Rating (CDR) and CDR-Sum of Box (CDR-SB), the Geriatric Depression Scale (GDS), and the Neuropsychiatric Inventory Questionnaire (NPI-Q) were used. To evaluate cognitive impairment, neuropsychological measures including the MMSE total score for global cognition and the composite scores for memory (ADNI-Mem) and executive function (ADNI-EF) were used [29, 30]. The neuropsychological measures at the 1-year and 2-year follow-up including MMSE, ADNI-Mem and ADNI-EF were also used for longitudinal analysis.

### MR image processing and subtype classification algorithm

Baseline T1-weighted images obtained using a standardized 1.5 Tesla MRI protocol (http://adni.loni.usc.edu/methods/mri-analysis/mri-acquisition/) of the ADNI-1 study were used. Details of the MRI acquisition and processing method have been previously described [31]. All T1-weighted MR images used in this study were automatically segmented using FreeSurfer version 5.1 (http://surfer.nmr.mgh.harvard.edu/) to perform regional volumetric measurements on the hippocampus, intracranium and cerebral cortices based on the Desikan-Killiany atlas [32]. After visual inspection and manual correction of segmentation errors, 5 CN and 8 AD cases were excluded because of poor-quality of segmentation. As such, 222 CN and 163 AD subjects were included in the present study.

The classification algorithm is described below. First, hippocampal and cortical regions-of-interest (ROI) were defined based on regions used in the previous studies in which NFT were counted for neuropathological classification of AD [20, 21]: (1) hippocampal ROI as the sum of the bilateral hippocampi, and (2) three cortical ROIs including frontal ROI as the sum of the bilateral middle frontal cortices, temporal ROI as the sum of the bilateral superior temporal cortices and parietal ROI as the sum of the bilateral inferior parietal cortices. The raw volume of each ROI was scaled by the intracranial volume (ICV) to obtain normalized volumes adjusted for individual head size. Second, age- and gender-specific norms of normalized ROI volumes were obtained from the overall CN dataset by constructing a norm table (S1 Table) as described by previous studies [33–39]. We stratified overall CN group into four overlapping age strata and two gender strata, as it has been previously reported that both age and gender potentially affect brain atrophy [23, 24]. Age subgroups were defined by overlapping stratification method that has been widely used to maximize the quantity of information obtained from normative data in previous studies [33–39]. Details of constructing the norm table are described in S1 File. Next, the Z-scores of hippocampal ROI (Z_H_), frontal (Z_F_), temporal (Z_T_) and parietal (Z_P_) ROI volumes of AD subjects were calculated based on age- and gender-specific norms. The cut-off value of Z < -1.0 as the determinant of the presence of prominent atrophy in each ROI was chosen based on the previous study [26, 40]. Finally, using the classification algorithm described in Table 1, all AD subjects were assigned to one of the following subtypes: (i) both impaired (BI), (ii) hippocampal atrophy only (HA), (iii) cortical atrophy only (CA), and (iv) both spared (BS).

**Table 1:** Classification algorithm based on regional atrophy patterns and definition of subtypes.

Subtypes	Hippocampal ROI Z-score (Z_H_)	Cortical ROIs Z-scores (Z_F_, Z_T_, Z_P_)
**Both impaired (BI)**	Z_H_ < -1.0	at least 1 of 3 cortical ROIs Z-scores < -1.0
**Hippocampal atrophy only (HA)**	Z_H_ < -1.0	all cortical ROIs Z-scores ≥ -1.0
**Cortical atrophy only (CA)**	Z_H_ ≥ -1.0	at least 1 of 3 cortical ROIs Z-scores < -1.0
**Both spared (BS)**	Z_H_ ≥ -1.0	all cortical ROIs Z-scores ≥ -1.0

ROI, Region-of-interest; Z_H_, Z-score of hippocampal ROI volume; Z_F_, Z-score of frontal ROI volume; Z_T_, Z-score of temporal ROI volume; Z_P_, Z-score of parietal ROI volume.

### Voxel-based morphometry (VBM)

VBM analysis was performed using SPM8 (http://www.fil.ion.ucl.ac.uk/spm/) with the VBM8 toolbox (http://dbm.neuro.uni-jena.de/vbm/) to demonstrate the overall regional gray matter (GM) atrophy pattern for each subtype. All T1-weighted images of each subject were normalized into standard anatomical space using a linear 12-parameter affine transformation with a customized template created from all images of CN and AD subjects as the reference. Normalized images were next segmented into GM, white matter, and CSF. After segmentation and modulation, smoothing at 8 mm full width-at-half maximum was performed.

### CSF biomarker analysis

Details of the CSF collection methods and CSF Aβ_1–42_, t-tau, and p-tau measurements are provided in the ADNI procedural manual and were described in a previous study [41]. The xMAP Luminex platform and Innogenetics/Fujirebio AlzBio3 Research Use Only immunoassay kits were used for CSF biomarker analysis. The cut-off values for CSF Aβ-positive (Aβ_1–42_ ≤ 192 pg/mL) were based on the previous studies [41, 42].

### Statistical analysis

For cross-sectional comparison of demographic and clinical variables, we performed analysis of variance (ANOVA) for continuous variables with post-hoc test and chi-square test for categorical variables (*p* < 0.05). Analysis of covariance (ANCOVA) was used to compare cross-sectional neuropsychological measures with educational levels as a covariate. On VBM analysis, comparisons between CN and each AD subtype were achieved with age (continuous variable) and gender as nuisance covariates at the *p* < 0.05 (*k* = 100) level after family-wise error correction. For CSF biomarker analysis, Kruskal-Wallis test and Mann-Whitney *U*-test was used. Linear mixed model (LMM) analyses with random intercepts were performed to examine the effect of subtype-by-time interaction on the longitudinal change in neuropsychological measures over 2 years in AD subjects. The fixed-effect factors were subtype, time, and the subtype-by-time interaction; the random effect factor was the participants. Educational level was controlled in the mixed models. All statistical analyses were performed using SPSS software version 21.0.

## Results

### Frequency of AD subtypes

Based on the classification algorithm, the BI subtype was the most frequent (58.9%) and the HA subtype the second most frequent (19.0%). The CA and BS subtypes accounted for 11.7% and 10.4% of AD subjects, respectively (Table 2).

**Table 2:** Mean normalized ROI volumes and Z-scores of AD subtypes.

	BI (n = 96: 58.9%)	HA (n = 31: 19.0%)	CA (n = 19: 11.7%)	BS (n = 17: 10.4%)
***Normalized ROI volumes***				
Hippocampal ROI	0.0033 (0.0004)	0.0034 (0.0004)	0.0046 (0.0007)	0.0045 (0.0006)
Frontal ROI	0.0217 (0.0022)	0.0240(0.0014)	0.0211 (0.0025)	0.0243 (0.0021)
Temporal ROI	0.0112 (0.0012)	0.0128 (0.0008)	0.0117 (0.0013)	0.0130 (0.0013)
Parietal ROI	0.0127 (0.0015)	0.0150 (0.0010)	0.0122 (0.0022)	0.0154 (0.0016)
***Z-score***				
Hippocampal ROI	-2.20 (0.73)	-2.13 (0.76)	-0.20 (1.06)	-0.15 (0.69)
Frontal ROI	-1.05 (1.02)	-0.04 (0.61)	-1.40 (1.16)	0.24 (0.91)
Temporal ROI	-1.38 (0.90)	-0.22 (0.57)	-1.06 (1.00)	0.14 (0.86)
Parietal ROI	-1.52 (0.96)	-0.21 (0.64)	-1.92 (1.39)	0.23 (0.92)

Data are presented as mean (SD). ROI, Region-of-interest; AD, Alzheimer’s disease; BI, Both impaired; HA, Hippocampal atrophy only; CA, Cortical atrophy only; BS, Both spared.

### Baseline demographic, clinical, and neuropsychological characteristics

There was a significant group difference in educational level, but not in age and gender, among the AD subtypes (Table 3). The frequency of ApoE ε4 allele carriers did not show significant group difference among the AD subtypes. No significant group differences were found in age at onset, frequency of subjects whose age at onset < 65 years, as well as disease duration across AD subtypes. Most baseline clinical variables including global CDR, GDS and NPI-Q did not show significant group differences among the AD subtypes, although the BS had relatively lower CDR-SB compared to the BI. Compared to CN, all AD subtypes showed significantly greater cognitive impairments in all neuropsychological measures. When comparing MMSE and ADNI-Mem among the AD subtypes, the BI had the lowest scores and the BS showed relatively less impairment compared to the BI. In terms of ADNI-EF, the CA exhibited the worst score, whereas the HA and the BS showed less impairment compared to the CA.

**Table 3:** Baseline demographic, clinical characteristics and neuropsychological measures of subjects.

	CN (n = 222)	AD (n = 163)				*p*
		BI (n = 96)	HA (n = 31)	CA (n = 19)	BS (n = 17)	AD subtypes^a^
***Demographic characteristics***						
Age	75.8 (5.0)	75.7 (7.4)	74.6 (7.1)	72.0 (8.9)	78.6 (7.2)	0.062
Female	107 (48%)	41 (43%)	21 (68%)	10 (53%)	7 (41%)	0.095
Education (years)	16.1 (2.8)	15.0 (2.9)	13.5 (2.8)	16.1 (3.2)	14.0 (2.9)	0.010^b^
ApoE ε4 carriers	60 (27%)	67 (70%)	21 (68%)	8 (42%)	9 (53%)	0.091
***Clinical characteristics at baseline***						
Age at onset^c^		72.3 (8.0)	71.1 (6.9)	69.6 (9.5)	75.8 (8.1)	0.129
Age at onset < 65years^c^		16 (17.4%)	5 (17.4%)	5 (26.3%)	1 (6.3%)	0.476
Disease duration (years) ^c^		3.39 (2.32)	3.46 (2.87)	2.38 (1.79)	2.56 (1.89)	0.219
CDR domain						
Memory	0 (0)	1.03 (0.34)	1.05 (0.35)	0.89 (0.46)	0.82 (0.25)	0.069
Orientation	0 (0)	0.85 (0.33)	0.89 (0.38)	0.61 (0.49)	0.62 (0.33)	0.004^d^
Judgment	0.03 (0.12)	0.81 (0.36)	0.71 (0.25)	0.84 (0.37)	0.62 (0.33)	0.098
Community affairs	0 (0.03)	0.73 (0.41)	0.77 (0.48)	0.58 (0.34)	0.56 (0.53)	0.189
Home and hobbies	0 (0)	0.80 (0.46)	0.82 (0.57)	0.71 (0.45)	0.59 (0.57)	0.358
Personal care	0 (0)	0.22 (0.44)	0.23 (0.43)	0.05 (0.23)	0.06 (0.24)	0.197
Global CDR	0 (0)	0.77 (0.25)	0.76 (0.25)	0.63 (0.23)	0.65 (0.23)	0.056
CDR-SB	0.03 (0.12)	4.44 (1.52)	4.47 (1.75)	3.68 (1.74)	3.26 (1.63)	0.016^e^
GDS	0.85 (1.15)	1.68 (1.46)	1.61 (1.50)	1.84 (1.61)	1.24 (0.83)	0.616
NPI-Q	0.36 (0.93)	3.60 (3.36)	3.55 (2.77)	3.00 (3.84)	3.65 (4.42)	0.915
***Neuropsychological measures at baseline***						
MMSE^f^	29.11 (1.00)	23.07 (2.08)	23.42 (1.98)	23.58 (1.57)	24.41 (2.48)	0.049^g^
ADNI-Mem^f^	0.97 (0.53)	-0.91 (0.51)	-0.74 (0.46)	-0.64 (0.58)	-0.45 (0.45)	0.001^g^
ADNI-EF^f^	0.70 (0.67)	-0.98 (0.75)	-0.50 (0.85)	-1.31 (1.01)	-0.53 (0.87)	0.001^h^

Data are shown as mean (SD) or n (%). CN, Cognitively normal; AD, Alzheimer’s disease; BI, Both impaired; HA, Hippocampal atrophy only; CA, Cortical atrophy only; BS, Both spared; CDR, Clinical dementia rating scale; CDR-SB, CDR-Sum of box; GDS, Geriatric depression scale; NPI-Q, Neuropsychiatric inventory questionnaire; MMSE, Mini-mental state examination; ADNI-Mem, composite score of memory function; ADNI-EF, composite score of executive function.

^a^ Analysis of variance (ANOVA), analysis of covariance (ANCOVA) and chi-square tests to test group differences among AD subtypes were performed.

^b^ The CA subtype had higher educational level than HA subtype in post-hoc test (*p* = 0.015).

^c^ Data for five AD subjects were not available (n = 92 for the BI, n = 16 for the BS).

^d^ The CA subtype showed lower CDR orientation domain score compared to the BI and HA subtypes (*p* = 0.041; *p* = 0.049, respectively).

^e^ The BS subtype had significantly lower CDR-SB compared to the BI subtype (*p* = 0.036).

^f^ All AD subtypes showed significantly lower MMSE, ADNI-Mem, and ADNI-EF score compared to CN (*p* < 0.001).

^g^ Significant difference in MMSE and ADNI-Mem score was observed between the BI and the BS subtypes (*p* = 0.041; *p* = 0.002, respectively).

^h^ The CA subtype had significantly greater impairments in ADNI-EF score than the BS (*p* = 0.015) and the HA subtypes (*p* = 0.002). Compared to the BI subtype, the HA subtype showed less impairment in ADNI-EF score (*p* = 0.014).

### Overall patterns of GM atrophy at the whole-brain level in the AD subtypes

**Fig 1:**
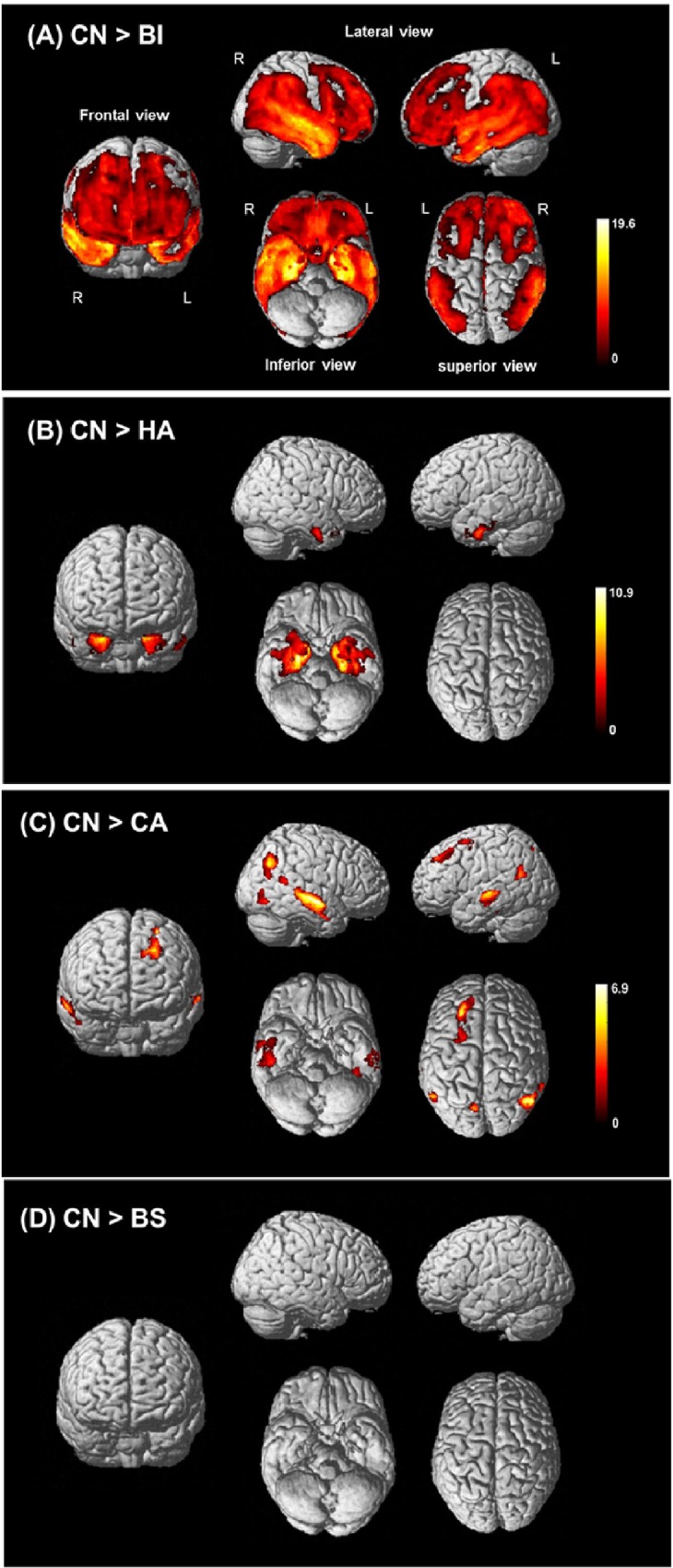
Overall regional brain atrophy pattern of AD subtypes in voxel-based morphometry. Voxel-wise whole-brain comparison of regional GM volume after correction for multiple comparisons using family-wise error correction at *p* < 0.05 (*k* = 100). (A) to (D) show the regional patterns of GM volume loss in each AD subtype compared with CN. (A) CN vs. BI, (B) CN vs. HA, (C) CN vs. CA and (D) CN vs. BS. GM, Gray matter; AD, Alzheimer’s disease; CN, Cognitively normal; BI, Both impaired; HA, Hippocampal atrophy only; CA, Cortical atrophy only; BS, Both spared.

To explore the overall regional GM atrophy pattern for each AD subtype, we conducted voxel-based analyses of the whole brain. As expected, the BI exhibited the most diffuse brain atrophy of all subtypes compared to CN, involving not only the bilateral temporal lobes but also the lateral fronto-parietal regions. The HA exhibited prominent atrophy only in the bilateral medial and inferior temporal regions including hippocampus. In contrast, the CA exhibited atrophy mainly on the lateral sides of the fronto-parieto-temporal cortices; both hippocampi were spared. The BS did not significantly differ in terms of GM volume compared to CN (Fig 1).

### Longitudinal courses of cognitive decline over 2 years

**Fig 2:**
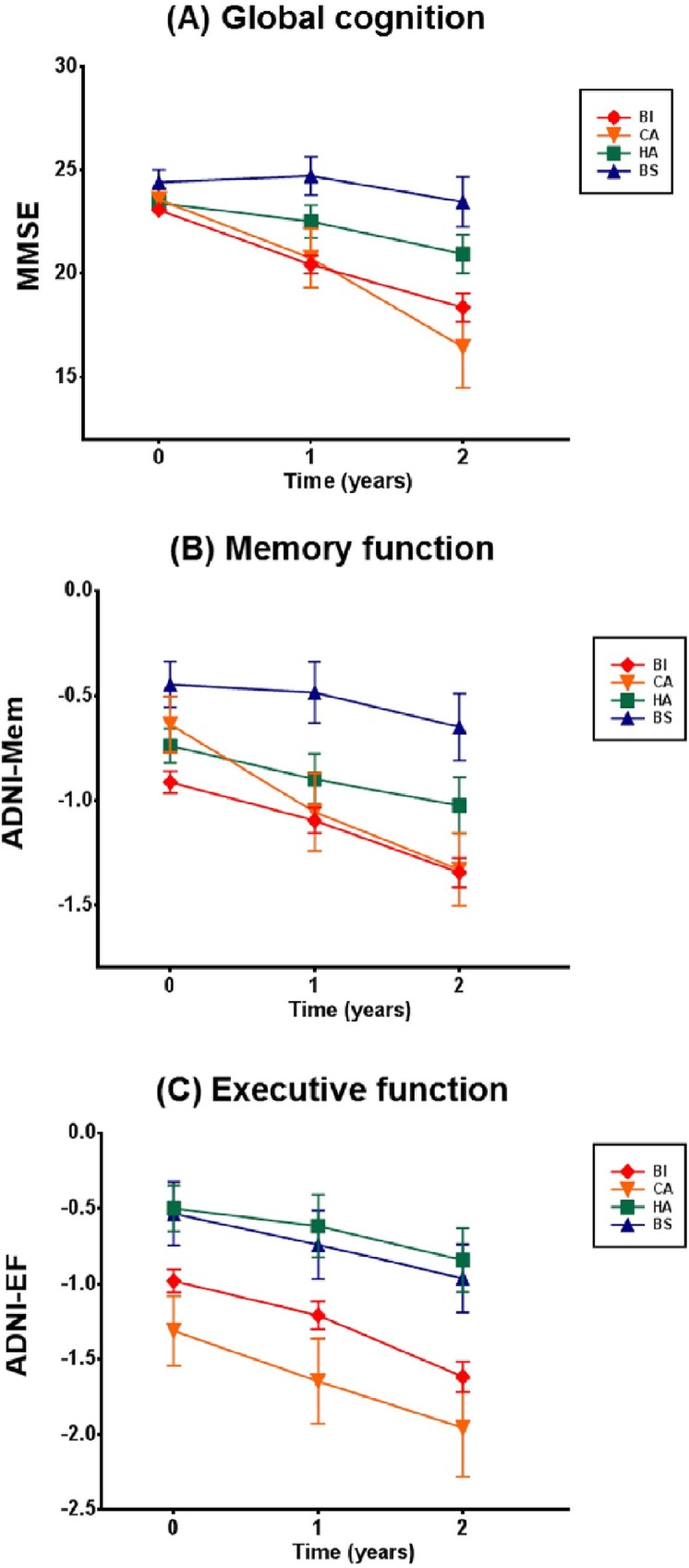
Longitudinal changes of cognitive function over 2 years across AD subtypes. Baseline, 1-year, and 2-year follow-up data on (A) MMSE indicating global cognition, (B) ADNI-Mem indicating memory function and (C) ADNI-EF indicating executive function are plotted, with means and standard errors. AD, Alzheimer’s disease; BI, Both impaired; HA, Hippocampal atrophy only; CA, Cortical atrophy only; BS, Both spared; MMSE; Mini-mental state examination; ADNI-Mem, composite score of memory function; ADNI-EF, composite score of executive function.

Upon LMM analyses of neuropsychological measures over 2 years in AD subjects, the subtype-by-time interaction, the subtype and time effects were all significant (S2 Table). Compared to the BI, the BS showed the slowest progression rates and the HA also showed relatively slow progression. In contrast, the CA showed the most rapid rates of cognitive decline over 2 years among the subtypes (Fig 2, S3 Table).

### CSF biomarkers

**Fig 3:**
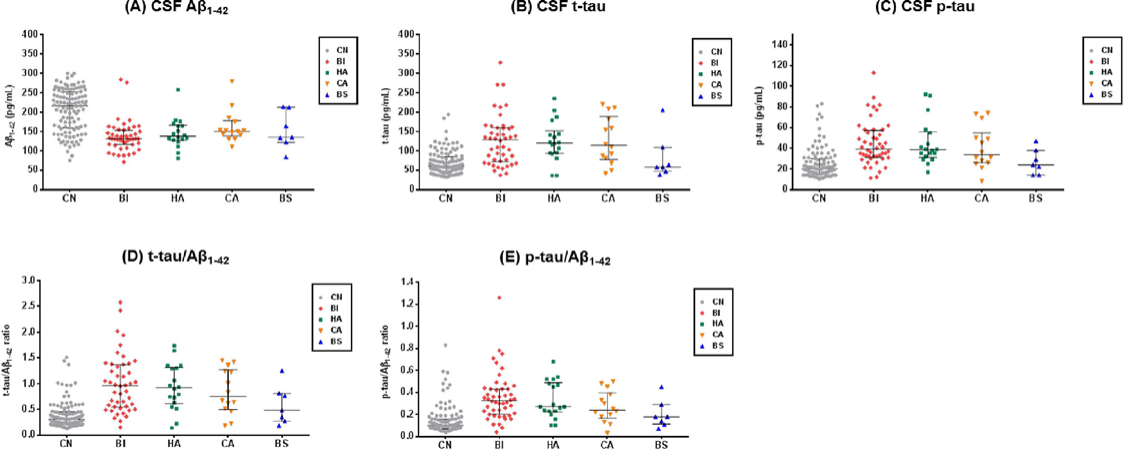
CSF biomarker levels. The dot plots of CSF biomarkers in CN and AD subtypes. Bars indicates median and IQR. (A) CSF Aβ_1–42_, (B) CSF t-tau, (C) CSF p-tau, (D) t-tau/Aβ_1–42_ ratio, (E) p-tau/Aβ_1–42_ ratio. CN, Cognitively normal; AD, Alzheimer’s disease; BI, Both impaired; HA, Hippocampal atrophy only; CA, Cortical atrophy only; BS, Both spared; CSF, Cerebrospinal fluid; Aβ_1–42_ = Amyloid-β 1–42 peptide; t-tau, total tau; p-tau, phosphorylated tau.

Of subjects for whom CSF data were available, all AD subtypes had significantly lower CSF Aβ_1–42_ levels compared to CN (Fig 3, Table 4). In terms of CSF tau level, the BI, HA and CA, but not BS, showed significant increase in t-tau, p-tau level, t-tau/Aβ_1–42_ and p-tau/Aβ_1–42_ ratio compared to CN. However, no significant group differences were found in all CSF biomarkers across the AD subtypes. In addition, all of the four AD subtypes were identified among the subgroup of CSF Aβ-positive AD subjects (n = 80), with relatively similar frequency (58%, 21%, 15%, and 6% for the BI, HA, CA and BS, respectively).

**Table 4:** CSF biomarkers of subjects in CN and AD subtypes.

	CN (n = 112)	AD (n = 87)				*p*
		BI (n = 48: 55%)	HA (n = 18: 21%)	CA (n = 14: 16%)	BS (n = 7: 8%)	AD subtypes^a^
**Aβ**_**1–42**_, pg/mL	216 (158–253)^b^	131 (116–153)^c^	138 (127–166)^c^	151 (138–178)^c^	135 (122–212)^c^	0.094
**t-tau**, pg/mL	61 (47–85)^b^	129 (72–160) ^d^ ^,^ ^e^	120 (94–152)^d^	114 (78–189)^d^	58 (47–109)	0.202
**p-tau**, pg/mL	20 (16–30)^b^	39 (31–57)^f^	39 (31–56)^f^	34 (26–55)^f^	24 (14–38)	0.116
**t-tau/Aβ**_**1–42**_	0.31 (0.21–0.45)^b^	0.96 (0.54–1.37)^e^ ^,^ ^g^	0.92 (0.61–1.31)^g^	0.75 (0.50–1.27)^g^	0.48 (0.27–0.81)	0.245
**p-tau/Aβ**_**1–42**_	0.10 (0.07–0.16)^b^	0.32 (0.20–0.43)^g^	0.27 (0.22–0.49)^g^	0.24 (0.17–0.40)^g^	0.18 (0.11–0.29)	0.109

Data are shown as median (IQR). CN, Cognitively normal; AD, Alzheimer’s disease; BI, Both impaired; HA, Hippocampal atrophy only; CA, Cortical atrophy only; BS, Both spared; CSF, Cerebrospinal fluid; Aβ_1–42_, Amyloid-β 1–42 peptide; t-tau, total tau; p-tau, phosphorylated tau.

^a ^Kruskal-Wallis test to test group differences among AD subtypes were performed.

^b^ All CSF biomarkers showed significant group differences across 5 groups including CN and 4 AD subtypes (Kruskal-Wallis test, *p* < 0.001).

^c^ Compared to CN, all AD subtypes showed significantly lower CSF Aβ_1–42_ level (Mann-Whitney U test, *p* < 0.001 for the BI and HA; p = 0.007 and p = 0.016 for the CA and BS, respectively).

^d ^The BI, HA and CA subtype showed significantly high CSF t-tau level compared to CN (Mann-Whitney U test, *p* < 0.001).

^e^ Data for t-tau and t-tau/Aβ_1–42_ ratio were not available for two subjects in the BI subtype.

^f ^Compared to CN, the BI, HA and CA subtypes showed significantly high CSF p-tau level (Mann-Whitney U test, *p* < 0.001 for the BI and HA; p = 0.001 for CA).

^g ^Compared to CN, the BI, HA and CA subtypes showed significantly high t-tau/Aβ_1–42_ and p-tau/Aβ_1–42_ ratio (Mann-Whitney U test, *p* < 0.001).

## Discussion

In this study, we identified four distinct subtypes of AD with heterogeneous patterns of regional atrophy using classification algorithm based on MRI-measured volume of hippocampus and cortical regions. To minimize the confounding effects of normal aging and gender on atrophy, age- and gender-specific norms of ICV-corrected hippocampal and cortical ROI volumes obtained from a large CN population were used. The frequency of each subtype was as follows: BI (58.9%), HA (19.0%), CA (11.7%), and BS (10.4%). As expected, VBM analysis demonstrated whole-brain-level differences in overall atrophy across the subtypes, indicating that regional atrophy patterns are heterogeneous in AD. The subtypes exhibited different progression rates over 2-years; and, all had significantly lower CSF Aβ_1–42_ levels compared with CN, suggesting the presence of Aβ neuropathology irrespective of the subtypes. To the best of our knowledge, this is the first study to identify subtypes of AD exhibiting heterogeneous regional brain atrophy patterns on MRI by applying age- and gender-specific norms of regional brain volumes obtained from a large CN population. In addition, this study is first to report CSF biomarker profiles for such subtypes.

Although many AD patients (59%) were of the BI subtype, with typical atrophy in both hippocampi and association cortices [6–9, 21], about 41% were of other subtypes with no prominent atrophy in either the hippocampus or association cortical regions. In particular, about 22% of AD patients did not exhibit typical hippocampal atrophy (i.e., CA, BS) and 29% of AD patients did not have the typical cortical atrophy (i.e., HA, BS). Moreover, about 10% of AD patients did not have any prominent atrophy in either hippocampal or association cortical regions when definition of atrophy in our study (Z-score < -1.0) was applied (i.e., BS). These findings suggest that MRI-based neuronal injury biomarkers, which have recently been incorporated into the NIA-AA diagnostic guidelines for AD [4, 5], may be limited in their potential to enhance the diagnostic accuracy of AD. Such heterogeneity issues should be considered when using MRI-based neuronal injury biomarkers for diagnostic purposes.

Given that brain atrophy evident on MRI indicates underlying neuronal loss caused by neuronal degeneration or injury [12], heterogeneity in regional atrophy patterns likely reflects variations in the anatomical specificity of neuronal degeneration in AD. Indeed, previous post-mortem pathological studies have found three neuropathological subtypes of AD based on the NFT distribution patterns: one typical and two atypical subtypes including hippocampal-sparing and limbic-predominant AD [20, 21]. The CA and HA subtypes identified in our study had atrophy patterns and frequency similar to atypical subtypes noted in those previous studies. However, the frequency of the typical subtype (i.e. BI) differed from previous studies; moreover, our current study identified the BS subtype, which has not been previously identified using neuropathology-based classifications. These discrepancies likely stem from differences in classification methods and subject characteristics. Previous studies defined atypical subtypes based on NFT distribution. However, we defined the subtypes based on in vivo MRI-measured atrophy, in efforts to identify the subtypes in real clinical practice where neuropathology-based approaches are rarely used. In addition, compared to previous studies that included AD patients at later, more severe stages of the disease at the time of death, the AD patients included in our study were at relatively early stages of disease (i.e., CDR 0.5–1). Moreover, by applying norm-based controls of the effects of aging and gender on atrophy, we were able to identify the BS subtype, which has not been described in previous studies.

Most clinical characteristics such as global CDR did not significantly differ across the subtypes at baseline. However, the subtypes with hippocampal atrophy (i.e., BI, HA) exhibited more severe impairment in the memory domain, whereas the subtypes with cortical atrophy (i.e., BI, CA) exhibited greater impairment in executive function. This result is consistent with previous findings that hippocampal-sparing AD is associated with more non-amnestic presentations and the dysexecutive phenotypes of AD with greater cortical thinning of the fronto-parietal cortical regions [20, 21, 43]. Thus, domain-specific cognitive impairment profiles may be useful to infer patient subtype when MRI is unavailable.

In contrast to the relatively similar baseline global CDR, the rates of cognitive decline over 2 years differed among AD subtypes. The BS had the slowest progression of all subtypes, suggesting an association between progression rate and the extent of atrophy at baseline. In contrast, the CA exhibited the most rapid cognitive decline of all subtypes. In addition, the subtypes lacking cortical atrophy (i.e., BS, HA) had slower progression rates than did the subtypes with cortical atrophy (i.e., BI, CA). This finding is consistent with previous studies that reported more rapid cognitive decline in hippocampal-sparing AD compared to typical or limbic-predominant AD [20]. Therefore, identification of AD subtype via quantitative reading of MRI may indicate the 2-year prognosis.

Low-level CSF Aβ_1–42_ is one of the two biomarkers of Aβ deposition in AD [4, 5]. In this study, all AD subtypes including the BS subtype had significantly lower CSF Aβ_1–42_ levels than did CN, indicating that brain Aβ deposition was comparable to that of typical AD irrespective of AD subtype. In terms of CSF neuronal injury biomarkers, the BS subtype did not exhibit significant elevations in t-tau and p-tau levels, indicating that the extent of atrophy and CSF tau levels were correlated. The CSF biomarker profile of the BS subtype might be in line with previous neuropathological studies that reported the presence of “plaque-only AD”, which were neuropathologically confirmed as AD, but having none or only very few neocortical NFT [44–46]. In a previous study, plaque-only AD patients tended to progress more slowly and less impaired compared to those of plaque and tangle AD [46], similar to clinical characteristics of the BS subtype in the present study. However, since sample size of the BS subtype used for CSF biomarker analysis was limited, further evaluation using amyloid/tau imaging or autopsy will be needed.

The observed “conflict” in the BS subtype between the cognitive impairments and the MRI-measured neuronal injury biomarker is not well-explained by the current pathophysiological model [12, 47, 48], which regards cognitive impairment as the end-result of pathophysiological AD processes preceded by MRI-measured atrophy. In addition, compared with CN, even the subtypes lacking hippocampal atrophy exhibited significant memory impairments whereas the subtypes lacking cortical atrophy exhibited significant executive dysfunction. These findings suggest that other brain abnormalities not mediated by specific regional atrophy, such as functional network abnormalities, may play important roles in the development of cognitive impairment in AD. A recent study showing that memory deficits in AD are more likely to be attributable to disruptions of intrinsic connectivity than to hippocampal atrophy [49] is in line with our results. Functional dysconnectivity such as default mode network (DMN) abnormalities has been consistently reported in AD; moreover, regions associated with DMN are reported to be susceptible to amyloid plaque deposition [50–52]. Further studies on functional network abnormalities among AD subtypes would help to identify the mechanisms underlying cognitive impairment, irrespective of the regional atrophy patterns.

Our study has several strengths. First, the use of age- and gender-specific norms obtained from a large CN population, recruited from a multi-center study like ADNI using the standardized protocols, enhanced classification accuracy by allowing us to adjust the effects of normal aging and gender differences on atrophy. Second, the clinical characteristics of the subtypes, including progression rates and cognitive impairment profiles, afford valuable information usable for diagnosis and prognosis in clinical practice. Moreover, exploration of the CSF biomarker profiles of each subtype enabled us to understand the underlying pathophysiology contributing to clinical and biological heterogeneity among the subtypes.

There are some limitations to our study. First, the sample sizes of the BS and CA subtypes were relatively small, particularly in terms of the CSF biomarker and longitudinal analyses, potentially limiting the statistical power. In addition, as amyloid or tau imaging data were not included, we could not directly measure and compare brain Aβ or tau distributions at the neuroanatomical level across the subtypes. However, based on the results of CSF biomarker analysis in our study, we can assume that amyloid PET tracer retention might be significantly increased in all AD subtypes since CSF Aβ_1–42_ levels showed high correlation with amyloid PET imaging [53, 54]. In contrast, the patterns of tau PET imaging tracer retention are expected to be different across AD subtypes and be similar to those from MRI-measured atrophy in our study, based on a previously reported significant correlation between tau tracer retention and brain atrophy [55]. Further studies with larger sample sizes of certain subtypes, using amyloid or tau imaging, would address these limitations.

In conclusion, we identified the subtypes of AD exhibiting heterogeneity in the regional patterns of atrophy on MRI, after controlling of the effect of aging and gender on atrophy using age- and gender-specific norms obtained from large CN population. These subtypes showed different patterns of cognitive decline over 2 years and the result of CSF biomarker analysis suggests the presence of AD-related neuropathology irrespective of the subtypes. Such heterogeneity of neuronal injury biomarkers and related heterogeneous progression patterns in AD should be considered in clinical trials of therapeutic interventions, as well as in clinical practice.

## Supporting Information

S1 File Supplement text for S1 Table. (PDF)

S1 Table Age- and gender-specific normative value of normalized ROI volumes in CN subjects. (PDF)

S2 Table Linear mixed model analyses of neuropsychological measures over 2 years in AD subjects. (PDF)

S3 Table Neuropsychological measures at 1-year and 2-year follow-up in CN and AD subjects. (PDF)
