# Supplementary material for: Heterogeneity of Regional Brain Atrophy Patterns Associated with Distinct Progression Rates in Alzheimer’s Disease
Source: PLoS One. 2015 Nov 30;10(11):e0142756. doi: 10.1371/journal.pone.0142756 (PMC4664412; doi:10.1371/journal.pone.0142756)
Supplement: S1 File — (PDF) [file pone.0142756.s001.pdf]

S1 File. Supplement text for S1 Table

Total CN group was divided into four overlapping age tables, separately for gender. Overlapping age tables with midpoint ages at 5-year intervals from 62 through 82 years were initially developed. The age range around each midpoint age was ± 7 years and the maximum of age (90 years) was included in the oldest age group. In addition, due to limited sample size of the youngest age subgroup (midpoint age 62 years), age subgroups with midpoint age 62 and 67 years were merged to age subgroup with midpoint 64.5 years with age range of ± 9.5 years. Then, means and SD of normalized ROI volumes were calculated for each cell. Means and SD values from four overlapping age subgroups with midpoint age 64.5, 72, 77, and 82 years were applied to subjects whose age ranged from 55-69, 70-74, 75-79, and 80-90 years, respectively.
