# Supplementary material for: Heterogeneity of Regional Brain Atrophy Patterns Associated with Distinct Progression Rates in Alzheimer’s Disease
Source: PLoS One. 2015 Nov 30;10(11):e0142756. doi: 10.1371/journal.pone.0142756 (PMC4664412; doi:10.1371/journal.pone.0142756)
Supplement: S1 Table — (PDF) [file pone.0142756.s002.pdf]

S1 Table. Age- and gender-specific normative value of normalized ROI volumes in CN subjects.

Age (years)		N	Normalized ROI volumes			
			**Hippocampal ROI**	**Frontal ROI**	**Temporal ROI**	**Parietal ROI**
**Male**	**55-69**	57	0.0048 (0.0006)	0.0242 (0.0021)	0.0130 (0.0012)	0.0155 (0.0016)
	**70-74**	88	0.0046 (0.0006)	0.0239 (0.0021)	0.0129 (0.0011)	0.0152 (0.0016)
	**75-79**	103	0.0045 (0.0006)	0.0238 (0.0021)	0.0128 (0.0012)	0.0151 (0.0017)
	**80-90**	58	0.0042 (0.0005)	0.0232 (0.0022)	0.0125 (0.0013)	0.0146 (0.0017)
**Female**	**55-69**	51	0.0050 (0.0007)	0.0247 (0.0020)	0.0137 (0.0015)	0.0159 (0.0016)
	**70-74**	85	0.0048 (0.0006)	0.0244 (0.0022)	0.0133 (0.0013)	0.0155 (0.0016)
	**75-79**	99	0.0047 (0.0006)	0.0243 (0.0022)	0.0132 (0.0013)	0.0154 (0.0016)
	**80-90**	56	0.0046 (0.0005)	0.0239 (0.0023)	0.0127 (0.0011)	0.0149 (0.0017)

Data are presented as mean (SD). ROI, Region-of-interest; CN, Cognitively normal.
