# Supplementary material for: Heterogeneity of Regional Brain Atrophy Patterns Associated with Distinct Progression Rates in Alzheimer’s Disease
Source: PLoS One. 2015 Nov 30;10(11):e0142756. doi: 10.1371/journal.pone.0142756 (PMC4664412; doi:10.1371/journal.pone.0142756)
Supplement: S2 Table — (PDF) [file pone.0142756.s003.pdf]

S2 Table. Linear mixed model analyses of neuropsychological measures over 2 years in AD subjects.

	**Subtype**		**Time**		**Subtype × Time**	
	F	***P***	***F***	***P***	***F***	***P***
MMSE[a]	8.145	< 0.001*	34.236	< 0.001*	3.959	0.001*
ADNI-Mem[a]	7.192	< 0.001*	45.615	< 0.001*	2.430	0.027*
ADNI-EF[a]	8.880	< 0.001*	42.031	< 0.001*	2.164	0.047*

MMSE; Mini-mental state examination; ADNI-Mem, composite score of memory function; ADNI-EF, composite score of executive function.

*$p < 0.05$

[a] All mixed models included educational level as a covariate.
