# Supplementary material for: Heterogeneity of Regional Brain Atrophy Patterns Associated with Distinct Progression Rates in Alzheimer’s Disease
Source: PLoS One. 2015 Nov 30;10(11):e0142756. doi: 10.1371/journal.pone.0142756 (PMC4664412; doi:10.1371/journal.pone.0142756)
Supplement: S3 Table — (PDF) [file pone.0142756.s004.pdf]

S3 Table. Neuropsychological measures at 1-year and 2-year follow-up in CN and AD subjects.

	CN[a]	AD				*p*
		BI	HA	CA	BS	AD subtype[b]
1-year follow-up[c]						
MMSE	29.18 (1.13)	20.44 (4.03)	22.52 (3.78)	20.75 (5.69)	24.71 (3.45)	0.001[d]
ADNI-Mem	1.03 (0.62)	-1.10 (0.57)	-0.90 (0.58)	-1.06 (0.75)	-0.49 (0.55)	0.002[e]
ADNI-EF	0.84 (0.71)	-1.21 (0.87)	-0.62 (0.99)	-1.65 (1.13)	-0.74 (0.86)	0.002[f]
2-year follow-up[g]						
MMSE	29.10 (1.12)	18.37 (5.57)	20.95 (4.26)	16.46 (7.11)	23.46 (4.35)	0.002[d]
ADNI-Mem	1.05 (0.62)	-1.35 (0.58)	-1.03 (0.62)	-1.33 (0.63)	-0.65 (0.58)	< 0.001[e]
ADNI-EF	0.82 (0.71)	-1.62 (0.82)	-0.84 (0.97)	-1.96 (1.13)	-0.96 (0.81)	< 0.001[h]

Data are shown as mean (SD). CN, Cognitively normal; AD, Alzheimer's disease; BI, Both impaired; HA, Hippocampal atrophy only; CA, Cortical atrophy only; BS, Both spared; MMSE; Mini-mental state examination; ADNI-Mem, composite score of memory function; ADNI-EF, composite score of executive function.

[a] Compared to CN, all AD subtypes had significantly lower MMSE, ADNI-Mem, ADNI-EF score at 1-year and 2-year follow-up ($p < 0.001$).

[b] ANCOVA test with educational level as a covariate were performed among the AD subtypes.

[c] Number of available data at 1-year follow-up for MMSE, ADNI-Mem and ADNI-EF (CN (n = 205), BI (n = 88), HA (n = 23), CA (n = 16), BS (n = 14))

[d] Compared to the BS, the CA and BI had significantly greater impairments in MMSE total score at 1-year follow-up ($p = 0.028$; $p = 0.002$, respectively) and at 2-year follow-up ($p = 0.007$; $p = 0.014$, respectively) in post-hoc test.

[e] Compared to the BS, the CA and BI had significantly greater impairments in ADNI-Mem score at 1-year follow-up ($p = 0.026$; $p = 0.002$, respectively) and at 2-year follow-up ($p = 0.011$; $p = 0.001$, respectively) in post-hoc test.

[f] The BI and CA had significantly lower ADNI-EF score compared to the HA ($p = 0.03$; $p = 0.003$, respectively) and the CA had significantly lower ADNI-EF score compared to the BS ($p = 0.031$) at 1-year follow-up in post-hoc test.

[g] Number of available data at 2-year follow-up for MMSE (CN (n = 197), BI (n = 67), HA (n = 21), CA (n = 13), BS (n = 13)); for ADNI-Mem (CN (n = 197), BI (n = 70), HA (n = 21), CA (n = 13), BS (n = 13)); for ADNI-EF

(CN (n = 196), BI (n = 64), HA (n = 21), CA (n = 12), BS (n = 13))

[h] The BI and CA had significantly lower ADNI-EF score compared to the HA (p = 0.002; p = 0.001, respectively) and the CA had significantly lower ADNI-EF score compared to the BS (p = 0.014) at 2-year follow-up in post-hoc test.
